# 

**Published:** 1900-06

**Authors:** 


### BOOKS RECEIVED.

A Pocket Text-Book of Chemistry and Physics. By Walton Martin, M. D., and William H. Rockwell, Jr., A. B., M. D., of the College of Physicians and Surgeons, New York. In one 12mo volume of 366 pages, with 137 illustrations Just ready. Cloth, $1.50, net. Flexible red leather, $2.00, net. Philadelphia and New York: Lea Brothers & Co.

A Text-Book of Medical Treatment of Diseases and Symptoms. For the use of students and practitioners of medicine. By Nestor Tirard, M. D., F. R. C. P., Professor of Principles and Practice of Medicine, King's College, London. Adapted to the U. S. Pharmacopeia by E. Quin Thornton, M. D., of Jefferson Medical College, Philadelphia. In one 8vo volume of 624 pages. Just ready. Cloth, $4.00, net. Philadelphia and New York: Lea Brothers & Co.

A Practical Treatise on the Sexual Disorders of the Male and Female. New (2d) edition. By Robert W. Taylor, M. D., Clinical Professor of Venereal Diseases in the College of Physicians and Surgeons, New York. In one handsome 8vo volume of 435 pages, with ninety-one illustrations and thirteen plates in colors and monochrome. Cloth, $3.00, net. Philadelphia and New York: Lea Brothers & Co.

The Treatment of Fractures. By Charles Locke Scudder, M. D., Surgeon to the Massachusetts General Hospital, Out-Patient Department; Assistant in Clinical and Operative Surgery in the Harvard Medical School; assisted by Frederick J. Colton, M. D. Large 8vo, pp. 432, with 585 illustrations. Philadelphia: W. B. Saunders. 1900. [Price, $4.50, net.]

Diseases of the Stomach, their Special Pathology, Diagnosis and Treatment, with sections on Anatomy, Physiology, Chemical and Microscopical examination of Stomach contents, Dietetics, Surgery of the Stomach, etc. By John C. Hemmeter, M. D., Professor in the Medical Department of the University of Maryland, Baltimore. With many original illustrations a number of which are in colors. Second edition, enlarged and revised. Octavo. 898 pages. Price, $6.00 net, cloth. Philadelphia. P. Blakiston's Son & Co., 1012 Walnut Street. 1900.

Essentials of Surgery, arranged in the form of Questions and Answers. Prepared especially for Students of Medicine By Edward Martin, A. M., M. D., Clinical Professor of Genito-urinary Diseases in the University of Pennsylvania. Illustrated. Seventh edition revised and enlarged: Price, $1.00 net. Philadelphia: W. B Saunders. 1900.

Essentials of Diagnosis, arranged in the form of Questions and Answers. Prepared especially for students of medicine. By Solomon Solis-Cohen, M D., Professor of Clinical Medicine and Therapeutics in the Philadelphia Polyclinic, etc., and Augustus A. Eshner, M. D., Professor of Clinical Medicine in the Philadelphia Polyclinic, etc. Illustrated Second edition, revised and enlarged. Philadelphia: W. B. Saunders. 1900. [Price, $1.00.

The Annual of Eclectic Medicine and Surgery. Edited by John V. Stevens, M. D. Volume VIII. Embracing the papers and proceedings of the various state eclectic medical societies for the years 1897 and 1898. Octavo, pp. 538. Cloth, price, $2.00. Cincinnati, O.: The Scudder Brothers Co., Publishers. 1900.

Manuel Complet de Gynécologie Médicale et Chirurgicale. Par A Lutaud, Professeur de Gynécologie, Médecin Adjoint de Saint Legare; etc., etc., etc. Nouvelle édition entièrement refondue. Contenant la technique opératoire complèt, et 607 figures dans le texte. Paris: A. Maloine, Editeur, 23-25 rue de l'École de Médecine. 1900.

Diseases of the Intestines. A Handbook for Practitioners and Students of Medicine. By Max Einhorn, M. D., Professor of Medicine at the New York Post-Graduate Medical School and Hospital, and Visiting Physician at the German Dispensary, New York. Small 8vo, pp. xiv.—391. New York: William Wood & Co. 1900.

